# Blood purification in critically ill patients: not enough, but still helpful

**DOI:** 10.1186/s13054-023-04638-9

**Published:** 2023-09-18

**Authors:** Federico Pappalardo, Astrid Cardinale, Nicoletta D’Ettore, Giulia Maj

**Affiliations:** Department of CardioThoracic and Vascular Anesthesia and Intensive Care, AO SS Antonio e Biagio e Cesare Arrigo, Alessandria, Italy

## Dear Editor,

We read with great interest the meta-analysis by Becker et al., (Efficacy of CytoSorb®: a systematic review and meta‑analysis. *Critical Care* 2023; 27(1):215). The authors have undoubtedly made great efforts to pull all these studies together, and the principal methodology used for the analysis was sound. However, we feel obliged to bring to your attention the conclusions drawn by this article.

The primary interpretation of the article was that “To date, there is no evidence for a positive effect of CytoSorb® on mortality across a variety of diagnoses.” Regardless of the high heterogeneity of the patient groups and their underlying pathologies, which make a pooled analysis at least very tricky, a meta-analysis is just replicating the methodological limitations of the studies evaluated by the researchers. Specifically, in regards to Cytosorb, many of the trials conducted in the earlier phase of clinical adoption were not suitably designed, with issues related to unblinding, lack of concealment, improper sample size estimations assuming implausibly large treatment effects, and the use of short-term surrogate endpoints instead of patient-centered outcomes. Moreover, most trials failed to predictively enrich the trial populations with patients that were more likely to respond to the given intervention and have not enabled mechanisms to understand whether patients actually responded (favorably or not) to the treatment.

The conclusions of this work raise several important questions. The first is whether mortality is an adequate marker to assess the efficacy of a therapy like Cytosorb. In our view, the device is not primarily aimed at reducing mortality causatively, but to act as an adjunctive measure in cases where standard of care alone does not lead to the desired stabilization of patients. Hence, mortality is likely not a suitable study endpoint to assess its efficacy. The hemoadsorber is intended as an adjunct therapy that allows for the institution of other interventions that may, in combination, contribute to preventing mortality. Indeed, recent studies have shown effective removal of circulating cytokines [1] and other deleterious materials from the blood [2, 3], and have demonstrated an association with lower than predicted rates of mortality in severely ill patients, when combined with other standard-of-care therapies [4, 5]. Yet, it is still the primary disease that has to be treated to impact on the patients outcome: as an example, rhabdomyolysis is ‘symptomatically’ treated by myoglobin removal, but limb ischemia/crush syndrome tells a different clinical scenario warranting specific therapy. Maybe removal of myoglobin (however accomplished) helps to reduce the severity of renal dysfunction and, eventually, aids quicker and better renal recovery. Another consideration is the optimal timing and duration of Cytosorb treatment: would clinicians start only when the patient is connected to a CRRT machine or should this specific treatment be implemented earlier? How can we provide an impact of the therapy on renal function if this is delivered only when CRRT is required?

Similarly, improvement of P/F ratio in ARDS (with or without VV ECMO) might turn into faster weaning from the ventilator and less lung injury; however, antibiotics (in bacterial pneumonia) are still paramount for patient survival! All ECMO trials have enrolled patients right after initiation of extracorporeal circulation: in clinical practise, our experience is that the timing for when Cytosorb is needed is extremely heterogenous, not necessarily at the beginning of ECMO. Not a single study has compared the duration of Cytosorb in respect of the duration of ECMO: if we assume that the extracorporeal circulation is driving the hyperinflammatory syndrome, this should be targeted concomitantly.

Another question regarding the second part of the article’s interpretation that there is no evidence “… that justifies its [Cytosorb] widespread use in intensive care medicine”. This is, in our opinion, poorly justified. If the concept of a therapy not being recommended for clinical use due to a lack of influence on mortality were consistently implemented for all medical devices and therapies, there would be virtually no treatment options for critically ill intensive-care patients. For example, recent meta-analyses in other fields of critical care have shown that interventions often seem to be ineffective [6]; a finding that should be carefully considered also for such paper.

On this point, the authors themselves acknowledge the limitations of their analyses. Overall, to date the clinical trial literature evaluating Cytosorb is limited in terms of amount and quality and carries a relevant risk of bias. More specifically, the significant heterogeneity of the studies included was highlighted as a key limitation, with attention drawn to some studies reporting low patient numbers and large effect sizes, and inconsistencies in the number of absorbers used and duration of therapy. Differing pathologies were also considered a likely confounder by the authors, emphasising that the right dose and timing for each patient should be key considerations for the future. Indeed, any use of the device, whether widespread or not, should follow the principle of proper patient selection, timing and dosing, which is as important for Cytosorb as it is for any therapeutic intervention.

It is only with such an individualized approach that potential risks, added complexity to care and costs of Cytosorb but also other given therapies can be justified.

Intensive care medicine is a very complex field in which generalizations and simplifications do not contribute to improving knowledge or patient care. ICU physicians should lead this innovation in the clinical research on critically ill patients.

## Data Availability

Not applicable.
